# Physical Activity and Cardiovascular Health

**DOI:** 10.1155/jdr/3341765

**Published:** 2026-03-31

**Authors:** Alexander Polyak, Alexander Lee, Martha Gulati

**Affiliations:** ^1^ Department of Medicine, Cedars-Sinai Medical Center, Los Angeles, California, USA, cedars-sinai.edu; ^2^ Department of Cardiology, Davis Women’s Heart Center, Houston Methodist DeBakey Heart and Vascular Center, Houston, Texas, USA; ^3^ The Baim Institute for Clinical Research, Boston, Massachusetts, USA

**Keywords:** cardiovascular disease, cardiovascular outcomes, physical activity

## Abstract

Cardiovascular disease (CVD) remains the leading global cause of mortality, with physical activity (PA) and inactivity playing a significant role in its prevalence and outcomes. PA has been shown to substantially reduce cardiovascular and cardiometabolic risk through mechanisms such as favorable cardiac remodeling, improved endothelial function, and enhanced metabolic regulation. This review explores the broad cardiovascular benefits of PA across both general and diabetic populations, highlighting its impact on reducing disease incidence, improving cardiac function, and lowering cardiovascular mortality. Various forms of PA—including aerobic, resistance, and flexibility and balance training—demonstrate differing but complementary roles in promoting cardiovascular health. Despite strong evidence and guideline endorsements, participation rates remain low, with fewer than a quarter of US adults meeting the minimum PA recommendations. Barriers to PA—personal, environmental, and health‐related—remain significant and underaddressed. Enhanced patient education, clinician engagement, and tailored intervention strategies are essential to improve adherence and outcomes. Overall, PA is a critical, evidence‐based strategy for reducing CVD risk.

## 1. Introduction

Cardiovascular disease (CVD) is the leading cause of death worldwide, accounting for 32% of global mortality in 2022 [1]. Physical activity (PA) can play an important role in both management and prevention of CVD and can improve many cardiovascular (CV) and cardiometabolic risk factors such as blood pressure, dyslipidemia, insulin sensitivity, and endothelial dysfunction [2–4]. PA can also play an important role in both management and prevention of Type 2 diabetes and CVD in diabetic patients [5, 6]. Among patients with Type 2 diabetes, CVD remains the leading cause of morbidity and mortality, affecting about a third [7]. As the prevalence of metabolic syndrome and Type 2 diabetes rises, the need to understand and decrease the associated CVD risks, including PA, has become an important focus in medical research and public health policy making [8, 9]. But despite the known benefits of PA on CV health, less than a quarter of adult Americans meet the recommended minimum PA guidelines per current recommendations [10]. This review focuses on different types of PA, CV benefits of activity in the general population and diabetic population, and barriers to meeting activity goals.

## 2. Cardiovascular Health

### 2.1. Mechanism of PA on Improving Cardiovascular Health

CV health is a concept that has become better defined in the last two decades. The American Heart Association (AHA) has established CV health as “Life′s Essential 8” which is a combination of (1) health behaviors including avoiding tobacco use, keeping body mass index (BMI) under 25 kg/m^2^, attaining adequate sleep, eating a heart healthy diet, in addition to engaging in regular PA with the recommendation for 150 min of moderate or 75 min of vigorous PA per week; (2) health risk factors including controlling cholesterol levels, blood pressure, and blood glucose [11, 12].

PA improves CV health primarily through three mechanisms. First, PA encourages beneficial cardiac adaptations through both structural and molecular pathways. Increased CV workload during PA stimulates myocardial hypertrophy and increased chamber volumes that ultimately enhance cardiac output [13]. At a molecular level, PA upregulates ATP‐sensitive potassium channels in cardiomyocytes and favors signaling pathways, such as the insulin‐like growth factor 1/P13K/Akt pathways, that promote cardiac growth, increase contractility, and thwart pathologic remodeling [14, 15].

Second, PA induces vascular development. Increased vascular demand during PA creates shearing stress on endothelial cells, stimulating nitric oxide (NO) production. As a strong vasodilator, NO reduces vascular inflammation and improves endothelial function [16]. On a cellular level, PA also induces mitochondrial biogenesis. Higher mitochondrial density results in improved energy production and decreased oxidative stress, reducing vascular aging [17].

Third, PA favorably shifts metabolic profiles. Participation in PA reduces atherosclerosis risk by encouraging healthier lipid profiles of higher high‐density lipoprotein (HDL), lower low‐density lipoprotein (LDL), and lower triglyceride levels [18]. Additionally, PA enhances insulin sensitivity and improves glucose metabolism, overall lowering risk of Type 2 diabetes [19, 20]. This is driven by processes at the molecular level, particularly when increased glucose influx into muscles promotes phosphorylation of key proteins, including glycogen synthase and TBC1D4 [21].

### 2.2. Impact of PA on Cardiovascular Outcomes

There is substantial evidence supporting the beneficial impact of PA on CV outcomes [22]. In fact, the multisociety AHA/American College of Cardiology (ACC) guideline on prevention of CVD, management of hypertension, management of chronic coronary disease, and management of heart failure all have PA as a Class 1 recommendation (Table 1) [24–27]. Similar findings have been repeated even in longitudinal studies, such as the Framingham Heart Study, where PA is associated with lower all‐cause and CVD‐attributable death [28].

**Table 1:** Summary of recommendations for physical activity on cardiovascular outcomes.

Guideline	Physical activity recommendations to improve CVD	COR	LOE
2019 AHA/ACC Guideline for Primary Prevention of Cardiovascular Disease	Adults should engage in at least 150 min per week of accumulated moderate‐intensity or 75 min per week of vigorous‐intensity aerobic physical activity (or combination) to reduce ASCVD risk.	1	B‐NR
	For adults unable to meet the minimum recommendations activity recommendations, engaging in some moderate‐or‐vigorous‐intensity physical activity, even if less than the recommended amount, can be beneficial to reduce ASCVD risk.	IIa	B‐NR
2017 AHA/ACC Guideline for Management of Patients with Hypertension	Increased physical activity with a structured exercise program is recommended for adults with elevated blood pressure or hypertension.	1	A
2023 AHA/ACC Guideline for Management of Patients with Chronic Coronary Disease	For patients with CCD who do not have contraindications, an exercise regimen is recommended, including ≥ 150 min/week of moderate‐intensity or ≥ 75 min/week of higher intensity aerobic activities to improve functional capacity and QOL and to reduce hospital admission and mortality rates.	1	A
	For patients with CCD who do not have contraindications, resistance training exercises are recommended on ≥ 2 days a week to improve muscle strength, functional capacity, and cardiovascular risk factor control.	1	B‐R
2022 AHA/ACC Guideline for Management of Patients with Heart Failure	In the general population, healthy lifestyle habits such as regular physical activity, maintaining normal weight, healthy diet patterns, and avoiding smoking are helpful to reduce future risk of heart failure.	1	B‐NR

*Note:* The COR refers to the strength of the recommendation in terms of the certainty of benefit compared to the proportion of risk, while the LOE rates the quality of evidence supporting the recommendation in terms of type, quantity, and consistency of data from research [23].

Abbreviations: ACC American College of Cardiology; AHA, American Heart Association; ASCVD, atherosclerotic cardiovascular disease; CCD, chronic coronary disease; COR, class of recommendation; LOE, level of evidence; QOL, quality of life.

## 3. PA

### 3.1. PA and Exercise

PA is any movement by a person that requires any amount of skeletal muscle movement leading to energy expenditure, such as everyday tasks including walking, work activity, leisure activity, cleaning, as well as structured movement such as exercise [29]. PA is quantified using the continuous metric of metabolic equivalent of task (MET) where one MET is defined as the energy used at rest or one′s basal metabolic expenditure, so PA with a MET value of 3 means using three times more energy than being still [30].

Exercise is a subset of PA that is usually planned and structured with the goal to increase or sustain fitness level [31]. Exercise can be grouped into categories—aerobic, resistance, and other (stretching, flexibility, balance) as summarized in Table 2 [5]. Aerobic activity is defined as continuous, rhythmic movements of large muscle groups, usually for at least 10 min at a time [32]. Examples of aerobic PA include walking, running, biking, or swimming—exercises that typically put the most demand on the CV and pulmonary systems [32]. Aerobic activity can be further stratified into light intensity (MET value 1.5–3, such as walking around the house or work setting), moderate intensity (MET value 3–6, such as jogging), or vigorous intensity (MET value > 6, such as running, swimming, or any aerobic activity that leads to an increase in heart rate) [30]. Resistance activities are brief, repetitive motions with weights or resistance (including body weight resistance) to improve muscle mass and endurance [32]. Resistance activities are usually MET value greater than 2 [4]. The last category of exercise includes stretching, flexibility, and balance. These types of exercises typically are a MET value of 2–4 and can improve joint mobility to maintain regular PA levels [19, 30]. Combining different forms of exercise has been shown to be most beneficial for overall cardiometabolic and CV health [33, 34].

**Table 2:** Summary of different exercises.

Type of exercise	METs	Examples
Aerobic—light intensity	1.5–3	Walking in the work setting, light housework
Aerobic—moderate intensity	3–6	Fast walking, light jogging, biking slowly
Aerobic—vigorous intensity	≥ 6	Running, swimming, biking
Resistance	≥ 2	Lifting weights, resistance bands
Other	2–4	Stretching, flexibility exercises, balance work

Abbreviation: METs, metabolic equivalent of task.

### 3.2. Physical Inactivity

Physical inactivity refers to when a person is not meeting their weekly PA recommendations (150 min of moderate or 75 min of vigorous PA per week), usually due to increased stationary activity with minimal energy expenditure such as sitting at a desk, watching television, or lying around while awake [35, 36]. The Sedentary Behavior Research Network defined sedentary behavior as “any waking behavior characterized by an energy expenditure less than 1.5 METs while in a sitting, lying, or reclining position” [36]. Healthcare providers tend to promote PA by prescribing exercise; however, it has been suggested that it may be more beneficial to emphasize not being inactive or sedentary [37]. A meta‐analysis found the largest benefit in terms of CVD risk reduction in diabetic patients to be when moving from total inactivity (0 METs) to some activity (6 MET hours per week), indicating possible “diminishing returns” on increasing PA duration [2]. The AHA guidelines for primary prevention of CVD have a Class IIb recommendation that decreasing sedentary behavior may reduce CVD risk [25]. The US Department of Health PA guidelines state that sedentary behavior is strongly correlated with CV mortality; however, there is not yet sufficient evidence to offer guidelines on exact recommended daily sedentary time [38].

### 3.3. Sex Differences in Cardiovascular Benefits of PA

Women have been shown to participate in PA less than men from early adolescence and continuing into adulthood [39]. An analysis in *The Lancet Global Health* from 2018 found that 31.7% of women are inactive compared to 23.4% of men representing a persistent “gender gap” in PA [40]. Prior studies have established differences between men and women in terms of physiologic response and exercise capacity, suggesting there may be sex differences for the CV benefits of PA [41, 42]. A meta‐analysis found a significant association by sex between PA and coronary heart disease, suggesting women had greater benefit of PA compared to men [43]. A recent prospective study from 2024 found that although men and women both achieved peak survival benefit at 300 min of aerobic exercise weekly, women had a significantly greater mortality benefit compared to men for the same amount of exercise (24% vs. 18%; *p* < 0.001) [42]. In addition, women who engaged in regular resistance training had a significantly increased mortality risk reduction compared to men (11% vs. 19%, *p* = 0.005) [42]. Interestingly, women derived the greatest mortality benefit with strength training once weekly, compared to men who had greatest benefit with strength training three times weekly [42]. Another study by Qian et al. analyzed what time of day exercise is most beneficial and found that men who exercised in the morning had the highest cardiorespiratory fitness, while women had the highest fitness in the evening group [44]. Sex‐specific risk factors, such as age at menarche, also need to be considered when assessing CVD in women [45]. Current recommendations for PA for the benefit of CV health do not differ by sex [46]; however, future guidelines may benefit from sex‐specific considerations given these emerging data.

### 3.4. Current Recommendations

There are slightly differing recommendations on optimal PA including type of activity, duration, and intensity; however, there is consensus that any level of PA is better than none, and there are significant CV benefits at essentially every level of PA [47]. Participants in a recent study had the most efficient CVD incidence reduction with 250 min of moderate‐to‐vigorous PA weekly, but significant incidence reduction was noted with even 10–15 min weekly [48].

Most organizational guidelines have adopted a form of the US Department of Health PA Guidelines that recommend everyone get at least 150 min per week of moderate‐intensity aerobic activity and at least 2 days of moderate‐intensity resistance exercises (Table 3) [46]. Unfortunately, only 10% of Americans are aware of PA guidelines, and only 3% know the correct amount of aerobic exercise recommended, highlighting the importance of physician communication and prescription of exercise [50].

**Table 3:** How much physical activity is recommended [5, 24, 25, 27, 46, 49].

Guidelines	Physical activity recommendations
US Department of Health Physical Activity Guideline	At least 150 min of moderate‐intensity or 75 min of vigorous‐intensity activity with at least 2 days of muscle‐strengthening activity per week.
AHA/ACC Guideline for Primary Prevention of Cardiovascular Disease	At least 150 min per week of accumulated moderate‐intensity or 75 min per week of vigorous‐intensity aerobic physical activity
AHA/ACC Guideline for Management of Patients with Chronic Coronary Disease	At least 150 min per week of moderate‐intensity aerobic activities or at least 75 min per week of higher‐intensity aerobic activities. Resistance (strength) training exercises are also recommended on at least 2 days per week.
AHA/ACC Guideline for Management of Patients with Hypertension	Either or combination of aerobic exercise (90–150 min per week), dynamic resistance exercise (90–150 min per week), and isometric resistance exercise (three times per week).
American Diabetes Association	At least 150 min or more of moderate‐ to vigorous‐intensity activity weekly, spread over at least 3 days per week, with no more than 2 consecutive days without activity. Shorter durations (minimum 75 min/week) of vigorous‐intensity or interval training may be sufficient for younger and more physically fit individuals.
American College of Sports Medicine	Recommend moderate‐intensity aerobic physical activity for a minimum of 30 min on 5 days per week, or vigorous‐intensity aerobic activity for a minimum of 20 min on 3 days per week. Every adult should perform activities that maintain or increase muscular strength and endurance for a minimum of 2 days per week.

Abbreviations: ACC, American College of Cardiology; AHA, American Heart Association.

## 4. PA for Persons With Type 2 Diabetes

Type 2 diabetes is defined as hyperglycemia due to insulin resistance. Over 537 million people worldwide in 2021 had Type 2 diabetes, with prevalence expected to increase to 783 million people worldwide by 2045 [51]. CVD is a major complication of diabetes and is associated with increased risk of ischemic heart disease, heart failure, coronary artery disease, and peripheral artery disease [52]. People with Type 2 diabetes have over 50% higher risk of all‐cause mortality and CV mortality compared to people without diabetes [53]. PA improves CV health through three main mechanisms—encouraging beneficial structural and molecular cardiac adaptations, inducing vascular development, and favorably shifting metabolic profiles including improved glucose metabolism—making PA an important aspect of nonpharmacologic prevention and management of Type 2 diabetes [13, 16, 19, 20].

### 4.1. PA and the Prevention of Type 2 Diabetes

PA, including both aerobic and resistance exercises, is important as it can lead to reducing incidence of Type 2 diabetes and CVD [54]. The Coronary Artery Risk Development in Young Adults (CARDIA) study showed that less fit young adults, measured as lower CRF, were more likely to develop Type 2 diabetes in adulthood [55]. The Diabetes Prevention Study randomly assigned overweight adults to a control group or an intervention group that would focus on diet and exercise with a goal of 30 min a day of PA; they found that the intervention group had a 58% reduction in Type 2 diabetes incidence over the next 4 years compared to the control group [56]. Notably, the effect of PA was greater in the more sedentary population prior to intervention (< 7.5 MET hours per week at baseline) and remained significant when adjusted for weight [57].

Another meta‐analysis showed that just 1 MET increase in activity was associated with an 8% lower risk of Type 2 diabetes incidence [58]. A pooled analysis of prospective observational cohort studies of the general population found inactivity to be associated with a 24% higher risk of CVD and a 42% higher risk of Type 2 diabetes [59]. A sedentary lifestyle significantly affects our cardiometabolic health and increases the risk of type 2 diabetes incidence [35].

### 4.2. PA and Cardiovascular Outcomes in Diabetes

Prior studies have shown that PA can improve CV outcomes in people with diabetes. A meta‐analysis showed that PA in people with Type 2 diabetes was associated with a 29% lower risk of CV death compared to patients who were inactive [53]. A pooled analysis of 10 population‐based cohort studies showed that people with diabetes (Type 1 and Type 2) who met their PA goals had a 35% lower all‐cause mortality [60]. However, the research on PA for people with Type 1 diabetes compared to Type 2 diabetes is limited and warrants further investigation.

The Look AHEAD clinical trial examined lifestyle intervention on obese patients with Type 2 diabetes and did not find a significant reduction in CV events; however, a post hoc analysis showed patients who lost more than 10% of their body weight did have a significant reduction in CV events [61]. The role of weight loss in the benefits of PA is not as clear. Wahid et al. showed that PA improves CV risk factors but only noted a marginal change in CVD risk when adjusting for body weight, indicating that the CVD benefits of PA may be derived from mechanisms other than weight loss [2].

Cardiac function may also improve with PA in people with Type 2 diabetes. Type 2 diabetes has been shown to be associated with increased incidence of heart failure with subclinical systolic and diastolic dysfunction [62]. A meta‐analysis of six clinical trials found that PA in people with Type 2 diabetes can lead to improvement in early diastolic velocity and systolic function by echocardiography [62].

### 4.3. PA Recommendations for Persons With Diabetes

The American Diabetes Association and Diabetes Canada both have strong recommendations for PA, recommending at least 150 min or more of moderate‐to‐vigorous‐intensity activity weekly spread over at least 3 days per week, with no more than two consecutive days without activity. Meeting these PA recommendations has been shown to improve glycemic control and CV outcomes in people with diabetes [5, 32]. In fact, some of the most common comorbidities in people with Type 2 diabetes [63] that may increase CVD risk, including obesity, dyslipidemia, hypertension, and chronic kidney disease, all have governing societies that also have strong recommendations for PA (Table 4), further emphasizing the importance of PA for all persons [5, 19, 64–66].

**Table 4:** Recommendations for physical activity of common diabetic comorbidities.

Association/society	Recommendations for physical activity	Grade
American Diabetes Association	Adults should engage in 150 min or more of moderate‐ to vigorous‐intensity activity weekly, spread over at least 3 days per week, with no more than 2 consecutive days without activity. Shorter durations (minimum 75 min/week) of vigorous‐intensity or interval training may be sufficient for younger and more physically fit individuals.	A
Diabetes Canada		
European Association for the Study of Obesity	Advise an exercise training program based on 150 to 200 min of aerobic exercise at least at moderate intensity.	A
European Society of Cardiology Guidelines for the Management of Dyslipidemia	Recommend 3.5–7‐h moderately vigorous physical activity per week or 30–60 min most days.	A,B^a^
Kidney International Guidelines for Management of Chronic Kidney Disease	Advise moderate‐intensity physical activity for a cumulative duration of at least 150 min per week.	1D^b^

^a^Grade A recommendation for improvement in HDL (high‐density lipoprotein) and Class B recommendation for improvement in LDL (low‐density lipoprotein).

^b^Class I recommendation, but level of evidence is low (D).

## 5. Barriers to PA

**Figure 1:**
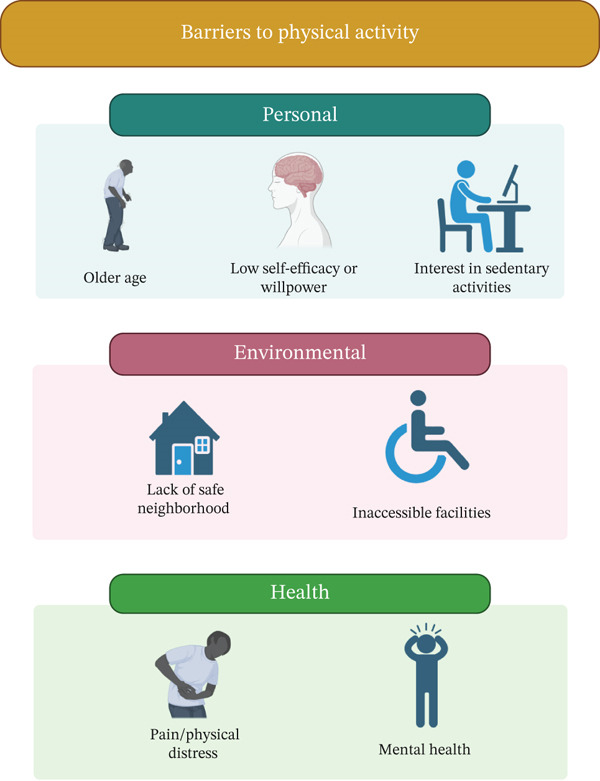
Barriers to physical activity. Figure summary—Different types of barriers can affect a person’s physical activity engagement including personal, environmental, and health‐related barriers. Identifying and understanding barriers to physical activity may improve a person′s adherence with physical activity.

Many different types of barriers (Figure 1) can affect a person′s PA participation including personal barriers (lack of time, energy, motivation, etc.), environmental barriers (access, cost, weather, etc.), and health‐related barriers (injury, fear of injury, chronic illness). Demographics that are at higher odds of reporting barriers to PA include those who are women, are older, earn a lower income, are married, have acquired higher levels of education, work full‐time jobs, and are smokers [67, 68]. The most prevalent barriers to PA among adults are low mood, lack of time, health issues, and lack of a partner [69]. Overcoming these barriers is vital so that individuals can meet their PA goals, and understanding the barriers is the first step for providers and patients to make individualized PA plans. The Center for Disease Control and Prevention (CDC) [70], the AHA [71], and CardioSmart [72] all provide online resources and recommendations for patients and providers to identify their specific barrier and help them to overcome these common barriers to PA.

### 5.1. Personal Barriers

Every individual′s unique personal life can impact PA participation. A person′s beliefs of their health impacts PA participation. People with lower self‐efficacy, lower willpower, or inappropriate goal setting are more likely to have lower levels of PA [68]. Similarly, people not yet in a stage of change mindset prove to be another barrier to PA engagement [68]. Those with more social support, on the contrary, are more likely to participate in PA [73]. Lastly, personal interests in sedentary activities, such as video games and watching television, are also reported barriers to PA [74]. Some suggested approaches to overcome personal barriers include identifying open times in a daily schedule to use for PA, making PA part of a daily routine, and choosing activities that can be done with friends to improve participation [70–72].

### 5.2. Environmental Barriers

An individual′s environment can heavily impact their ability to engage in PA. Insufficient access to places to exercise or safe neighborhoods to walk is a commonly reported barrier [19, 67]. Poor weather can deter people from PA, particularly outdoors [75]. In patients with Type 2 diabetes and hypertension, a study identified “crowded sidewalks, lack of green spaces, lack of proper lighting in public spaces, and dense traffic” as barriers to PA [76]. Accessibility and the logistics of partaking in PA become even more intricate in patients who have highly morbid conditions. For instance, patients with multiple sclerosis report that they are unable to participate in PA due to inaccessibility and fear of disability stigma in PA spaces [77]. Suggested approaches to overcome environmental barriers include selecting activities that require minimal facilities, are inexpensive and convenient, and can be done regardless of weather [70–72].

### 5.3. Health‐Related Barriers

Both physical and mental components of health impact an individual′s willingness and ability to participate in PA. Patients in physical discomfort or stress often report that their palpable symptoms are significant barriers to PA [76]. To highlight a few populations, in rheumatic and musculoskeletal disease patients, fatigue and swollen joints are the most commonly reported barriers [78]. In patients with Charcot‐Marie‐Tooth disease, difficulty with balance, pain, and poor muscle strength are frequent barriers [79].

Patients reporting mental health disorders are correlated with higher rates of sedentary behavior on average [80]. However, the relationship between mental health and PA is nuanced and complex. While many patients report symptoms like anxiety as a barrier to engaging in PA, other patients with anxiety were paradoxically more motivated to partake in PA due to its established benefits on mental health outcomes [80]. Suggested approaches to overcome health‐related barriers include talking with a healthcare provider to learn exercises that are appropriate for age, fitness, health, and skill level for the individual and starting slow to gain confidence with those specific exercises [70–72].

## 6. Future Directions and Conclusion

PA clearly has substantial evidence supporting its benefit in CVD outcomes, including in people with diabetes and other comorbid conditions. Yet, it ironically has remarkably low rates of participation among Americans. It is encouraging that there are several ongoing statements and efforts from multiple societies, ranging from the American Diabetes Association to the American College of Sports Medicine to the Physical Activity Guidelines Advisory Committee, promoting PA. The future direction of improving PA participation includes addressing barriers to PA, improving patient and provider education about the benefits of PA, and endorsing changes in public policy to encourage a more active American lifestyle.

## Funding

M.G.: This work was supported by contracts from the National Heart, Lung, and Blood Institute (Nos. N01‐HV‐068161, N01‐HV‐068162, N01‐HV‐068163, N01‐HV‐068164, Grants U01 HL064829, U01 HL649141, U01 HL649241, K23 HL105787, K23 HL125941, K23 HL127262, K23HL151867, T32 HL069751, R01 HL090957, R03 AG032631, R01 HL146158, R01 HL146158‐04S1, R01 HL124649, R01 HL153500, U54 AG065141); General Clinical Research Center grant MO1‐RR00425 from the National Center for Research Resources; the National Center for Advancing Translational Sciences grant UL1TR000124; Department of Defense grant PR161603 (CDMRP‐DoD); grants from the Gustavus and Louis Pfeiffer Research Foundation, Denville, New Jersey; the Women′s Guild of Cedars‐Sinai Medical Center, Los Angeles, California; the Ladies Hospital Aid Society of Western Pennsylvania, Pittsburgh, Pennsylvania; QMED, Inc., Laurence Harbor, New Jersey; the Edythe L. Broad and the Constance Austin Women′s Heart Research Fellowships, Cedars‐Sinai Medical Center, Los Angeles, California; the Barbra Streisand Women′s Cardiovascular Research and Education Program, Cedars‐Sinai Medical Center, Los Angeles, California; the Society for Women’s Health Research, Washington, DC; the Linda Joy Pollin Women′s Heart Health Program; the Erika Glazer Women′s Heart Health Project; the Adelson Family Foundation, Cedars‐Sinai Medical Center, Los Angeles, California; Robert NA. Winn Diversity in Clinical Trials Career Development Award (Winn CDA); and the Anita Dann Friedman Endowment in Women′s Cardiovascular Medicine & Research.

## Disclosure

This work is solely the responsibility of the authors and does not necessarily represent the official views of the National Heart, Lung, and Blood Institute, the National Institutes of Health, or the US Department of Health and Human Services. M.G. received consultant fees/honoraria from Medtronic, Novartis, and New Amsterdam.

## Conflicts of Interest

The authors declare no conflicts of interest.

## Data Availability

Data sharing not applicable to this article as no datasets were generated or analyzed during the current study.

## References

[1] Cardiovascular Diseases (CVDs) , published July 31, 2025, https://www.who.int/news-room/fact-sheets/detail/cardiovascular-diseases-(cvds).

[2] Wahid A. , Manek N. , Nichols M. , Kelly P. , Foster C. , Webster P. , Kaur A. , Smith C. F. , Wilkins E. , Rayner M. , Roberts N. , and Scarborough P. , Quantifying the Association Between Physical Activity and Cardiovascular Disease and Diabetes: A Systematic Review and Meta-Analysis, Journal of the American Heart Association. (2016) 5, no. 9, e002495, 10.1161/JAHA.115.002495, 2-s2.0-85017153698. 27628572 PMC5079002

[3] Lin X. , Zhang X. , Guo J. , Roberts C. K. , McKenzie S. , Wu W.-C. , Liu S. , and Song Y. , Effects of Exercise Training on Cardiorespiratory Fitness and Biomarkers of Cardiometabolic Health: A Systematic Review and Meta-Analysis of Randomized Controlled Trials, Journal of the American Heart Association. (2015) 4, no. 7, e002014, 10.1161/JAHA.115.002014, 2-s2.0-85016649825. 26116691 PMC4608087

[4] Dhuli K. , Naureen Z. , Medori M. C. , Fioretti F. , Caruso P. , Perrone M. A. , Nodari S. , Manganotti P. , Xhufi S. , Bushati M. , Bozo D. , Connelly S. T. , Herbst K. L. , and Bertelli M. , Physical Activity for Health, Journal of Preventive Medicine and Hygiene. (2022) 63, no. 2 Supplement 3, E150–E159, 10.15167/2421-4248/jpmh2022.63.2S3.2756. 36479484 PMC9710390

[5] Colberg S. R. , Sigal R. J. , Yardley J. E. , Riddell M. C. , Dunstan D. W. , Dempsey P. C. , Horton E. S. , Castorino K. , and Tate D. F. , Physical Activity/Exercise and Diabetes: A Position Statement of the American Diabetes Association, Diabetes Care. (2016) 39, no. 11, 2065–2079, 10.2337/dc16-1728, 2-s2.0-84994372704, 27926890. 27926890 PMC6908414

[6] Patel R. and Keyes D. , Lifestyle Modification for Diabetes and Heart Disease Prevention, StatPearls, 2024, StatPearls Publishing. 36251831

[7] Einarson T. R. , Acs A. , Ludwig C. , and Panton U. H. , Prevalence of Cardiovascular Disease in Type 2 Diabetes: A Systematic Literature Review of Scientific Evidence From Across the World in 2007-2017, Cardiovascular Diabetology. (2018) 17, no. 1, 10.1186/s12933-018-0728-6, 2-s2.0-85048255721. PMC5994068 29884191

[8] Seuring T. , Archangelidi O. , and Suhrcke M. , The Economic Costs of Type 2 Diabetes: A Global Systematic Review, PharmacoEconomics. (2015) 33, no. 8, 811–831, 10.1007/s40273-015-0268-9, 2-s2.0-84938420461, 25787932. 25787932 PMC4519633

[9] Bommer C. , Sagalova V. , Heesemann E. , Manne-Goehler J. , Atun R. , Bärnighausen T. , Davies J. , and Vollmer S. , Global Economic Burden of Diabetes in Adults: Projections From 2015 to 2030, Diabetes Care. (2018) 41, no. 5, 963–970, 10.2337/dc17-1962. 29475843

[10] Elgaddal N. , Kramarow E. A. , and Reuben C. , Physical Activity Among Adults Aged 18 and Over: United States, 2020, NCHS Data Brief. (2022) 443, 1–8. 36043905

[11] Lloyd-Jones D. M. , Hong Y. , Labarthe D. , Mozaffarian D. , Appel L. J. , Van Horn L. , Greenlund K. , Daniels S. , Nichol G. , Tomaselli G. F. , Arnett D. K. , Fonarow G. C. , Ho P. M. , Lauer M. S. , Masoudi F. A. , Robertson R. M. , Roger V. , Schwamm L. H. , Sorlie P. , Yancy C. W. , and Rosamond W. D. , Defining and Setting National Goals for Cardiovascular Health Promotion and Disease Reduction: The American Heart Association’s Strategic Impact Goal Through 2020 and Beyond, Circulation. (2010) 121, no. 4, 586–613, 10.1161/CIRCULATIONAHA.109.192703, 2-s2.0-76349121984, 20089546. 20089546

[12] Lloyd-Jones D. M. , Allen N. B. , Anderson C. A. M. , Black T. , Brewer L. C. , Foraker R. E. , Grandner M. A. , Lavretsky H. , Perak A. M. , Sharma G. , Rosamond W. , and American Heart Association , Life’s Essential 8: Updating and Enhancing the American Heart Association’s Construct of Cardiovascular Health: A Presidential Advisory From the American Heart Association, Circulation. (2022) 146, no. 5, e18–e43, 10.1161/CIR.0000000000001078, 35766027. 35766027 PMC10503546

[13] Wilson M. G. , Ellison G. M. , and Cable N. T. , Basic Science Behind the Cardiovascular Benefits of Exercise, British Journal of Sports Medicine. (2016) 50, no. 2, 93–99, 10.1136/bjsports-2014-306596rep, 2-s2.0-84954305606, 26729891. 26729891

[14] Vega R. B. , Konhilas J. P. , Kelly D. P. , and Leinwand L. A. , Molecular Mechanisms Underlying Cardiac Adaptation to Exercise, Cell Metabolism. (2017) 25, no. 5, 1012–1026, 10.1016/j.cmet.2017.04.025, 2-s2.0-85018999424, 28467921. 28467921 PMC5512429

[15] Wang X. and Fitts R. H. , Effects of Regular Exercise on Ventricular Myocyte Biomechanics and K_ATP_ channel function, American Journal of Physiology. Heart and Circulatory Physiology. (2018) 315, no. 4, H885–H896, 10.1152/ajpheart.00130.2018, 2-s2.0-85053835914, 30074836. 30074836

[16] Lobelo F. , Young D. R. , Sallis R. , Garber M. D. , Billinger S. A. , Duperly J. , Hutber A. , Pate R. R. , Thomas R. J. , Widlansky M. E. , McConnell M. V. , and Joy E. A. , Routine Assessment and Promotion of Physical Activity in Healthcare Settings: A Scientific Statement From the American Heart Association, Circulation. (2018) 137, no. 18, e495–e522, 10.1161/CIR.0000000000000559, 2-s2.0-85047507591, 29618598. 29618598

[17] Zhang X. and Gao F. , Exercise Improves Vascular Health: Role of Mitochondria, Free Radical Biology & Medicine. (2021) 177, 347–359, 10.1016/j.freeradbiomed.2021.11.002, 34748911. 34748911

[18] Bays H. E. , Jones P. H. , Brown W. V. , Jacobson T. A. , and National Lipid Association , National Lipid Association Annual Summary of Clinical Lipidology 2015, Journal of Clinical Lipidology. (2014) 8, no. 6, S1–S36, 10.1016/j.jacl.2014.10.002, 2-s2.0-84917730717. 25523435

[19] Kanaley J. A. , Colberg S. R. , Corcoran M. H. , Malin S. K. , Rodriguez N. R. , Crespo C. J. , Kirwan J. P. , and Zierath J. R. , Exercise/Physical Activity in Individuals with Type 2 Diabetes: A Consensus Statement from the American College of Sports Medicine, Medicine and Science in Sports and Exercise. (2022) 54, no. 2, 353–368, 10.1249/MSS.0000000000002800, 35029593. 35029593 PMC8802999

[20] Temple K. A. , Tjaden A. H. , Atkinson K. M. , Barengolts E. , Hannon T. S. , Mather K. J. , Utzschneider K. M. , Edelstein S. L. , Ehrmann D. A. , Mokhlesi B. , and RISE Consortium , Association of Habitual Daily Physical Activity With Glucose Tolerance and *β*-Cell Function in Adults With Impaired Glucose Tolerance or Recently Diagnosed Type 2 Diabetes From the Restoring Insulin Secretion (RISE) Study, Diabetes Care. (2019) 42, no. 8, 1521–1529, 10.2337/dc19-0538, 2-s2.0-85070182964, 31177181. 31177181 PMC6647043

[21] Sjøberg K. A. , Frøsig C. , Kjøbsted R. , Sylow L. , Kleinert M. , Betik A. C. , Shaw C. S. , Kiens B. , Wojtaszewski J. F. P. , Rattigan S. , Richter E. A. , and McConell G. K. , Exercise Increases Human Skeletal Muscle Insulin Sensitivity via Coordinated Increases in Microvascular Perfusion and Molecular Signaling, Diabetes. (2017) 66, no. 6, 1501–1510, 10.2337/db16-1327, 2-s2.0-85019545822, 28292969. 28292969

[22] Kunutsor S. K. and Laukkanen J. A. , Physical Activity, Exercise and Adverse Cardiovascular Outcomes in Individuals With Pre-Existing Cardiovascular Disease: A Narrative Review, Expert Review of Cardiovascular Therapy. (2024) 22, no. 1-3, 91–101, 10.1080/14779072.2024.2328644, 38488568. 38488568 PMC11057847

[23] Halperin J. L. , Levine G. N. , Al Khatib S. M. , Birtcher K. K. , Bozkurt B. , Brindis R. G. , Cigarroa J. E. , Curtis L. H. , Fleisher L. A. , Gentile F. , Gidding S. , Hlatky M. A. , Ikonomidis J. , Joglar J. , Pressler S. J. , and Wijeysundera D. N. , Further Evolution of the ACC/AHA Clinical Practice Guideline Recommendation Classification System: A Report of the American College of Cardiology/American Heart Association Task Force on Clinical Practice Guidelines, Circulation. (2016) 133, no. 14, 1426–1428, 10.1161/CIR.0000000000000312, 2-s2.0-84944346071, 26399660. 26399660

[24] Virani S. S. , Newby L. K. , Arnold S. V. , Bittner V. , Brewer L. C. , Demeter S. H. , Dixon D. L. , Fearon W. F. , Hess B. , Johnson H. M. , Kazi D. S. , Kolte D. , Kumbhani D. J. , LoFaso J. , Mahtta D. , Mark D. B. , Minissian M. , Navar A. M. , Patel A. R. , Piano M. R. , Rodriguez F. , Talbot A. W. , Taqueti V. R. , Thomas R. J. , van Diepen S. , Wiggins B. , Williams M. S. , and Peer Review Committee Members , 2023 AHA/ACC/ACCP/ASPC/NLA/PCNA Guideline for the Management of Patients With Chronic Coronary Disease: A Report of the American Heart Association/American College of Cardiology Joint Committee on Clinical Practice Guidelines, Circulation. (2023) 148, no. 9, e9–e119, 10.1161/CIR.0000000000001168, 37471501. 37471501

[25] Arnett D. K. , Blumenthal R. S. , Albert M. A. , Buroker A. B. , Goldberger Z. D. , Hahn E. J. , Himmelfarb C. D. , Khera A. , Lloyd-Jones D. , McEvoy J. W. , Michos E. D. , Miedema M. D. , Muñoz D. , Smith S. C. , Virani S. S. , Williams K. A. , Yeboah J. , and Ziaeian B. , 2019 ACC/AHA Guideline on the Primary Prevention of Cardiovascular Disease: A Report of the American College of Cardiology/American Heart Association Task Force on Clinical Practice Guidelines, Circulation. (2019) 140, no. 11, e596–e646, 10.1161/CIR.0000000000000678, 2-s2.0-85072058148, 30879355. 30879355 PMC7734661

[26] Heidenreich P. A. , Bozkurt B. , Aguilar D. , Allen L. A. , Byun J. J. , Colvin M. M. , Deswal A. , Drazner M. H. , Dunlay S. M. , Evers L. R. , Fang J. C. , Fedson S. E. , Fonarow G. C. , Hayek S. S. , Hernandez A. F. , Khazanie P. , Kittleson M. M. , Lee C. S. , Link M. S. , Milano C. A. , Nnacheta L. C. , Sandhu A. T. , Stevenson L. W. , Vardeny O. , Vest A. R. , and Yancy C. W. , 2022 AHA/ACC/HFSA Guideline for the Management of Heart Failure: Executive Summary: A Report of the American College of Cardiology/American Heart Association Joint Committee on Clinical Practice Guidelines, Circulation. (2022) 145, no. 18, e876–e894, 10.1161/CIR.0000000000001062, 35363500. 35363500

[27] Whelton P. K. , Carey R. M. , Aronow W. S. , Casey D. E. , Collins K. J. , Dennison Himmelfarb C. , DePalma S. M. , Gidding S. , Jamerson K. A. , Jones D. W. , Mac Laughlin E. J. , Muntner P. , Ovbiagele B. , Smith S. C. , Spencer C. C. , Stafford R. S. , Taler S. J. , Thomas R. J. , Williams K. A. , Williamson J. D. , and Wright J. T. , 2017 ACC/AHA/AAPA/ABC/ACPM/AGS/APhA/ASH/ASPC/NMA/PCNA Guideline for the Prevention, Detection, Evaluation, and Management of High Blood Pressure in Adults: Executive Summary: A Report of the American College of Cardiology/American Heart Association Task Force on Clinical Practice Guidelines, Hypertension. (2018) 71, no. 6, 1269–1324, 10.1161/HYP.0000000000000066, 2-s2.0-85054193365, 29133354. 29133354

[28] Shortreed S. M. , Peeters A. , and Forbes A. B. , Estimating the Effect of Long-Term Physical Activity on Cardiovascular Disease and Mortality: Evidence From the Framingham Heart Study, Heart. (2013) 99, no. 9, 649–654, 10.1136/heartjnl-2012-303461, 2-s2.0-84876101509, 23474622. 23474622

[29] Caspersen C. J. , Powell K. E. , and Christenson G. M. , Physical Activity, Exercise, and Physical Fitness: Definitions and Distinctions for Health-Related Research, Public Health Reports. (1985) 100, no. 2, 126–131, 3920711. 3920711 PMC1424733

[30] Harrington D. and Henson J. , Physical Activity and Exercise in the Management of Type 2 Diabetes: Where to Start?, Practical Diabetes. (2021) 38, no. 5, 35–40b, 10.1002/pdi.2361.

[31] Sigal R. J. , Kenny G. P. , Wasserman D. H. , and Castaneda-Sceppa C. , Physical Activity/Exercise and Type 2 Diabetes, Diabetes Care. (2004) 27, no. 10, 2518–2539, 10.2337/diacare.27.10.2518, 2-s2.0-4644320002, 15451933. 15451933

[32] Sigal R. J. , Armstrong M. J. , Bacon S. L. , Boulé N. G. , Dasgupta K. , Kenny G. P. , and Riddell M. C. , Physical Activity and Diabetes, Canadian Journal of Diabetes. (2018) 42, no. Supplement 1, S54–S63, 10.1016/j.jcjd.2017.10.008, 2-s2.0-85044793484. 29650112

[33] Marzolini S. , Oh P. I. , and Brooks D. , Effect of Combined Aerobic and Resistance Training Versus Aerobic Training Alone in Individuals With Coronary Artery Disease: A Meta-Analysis, European Journal of Preventive Cardiology. (2012) 19, no. 1, 81–94, 10.1177/1741826710393197, 2-s2.0-84856888525, 21450617. 21450617

[34] Liang M. , Pan Y. , Zhong T. , Zeng Y. , and Cheng A. S. K. , Effects of Aerobic, Resistance, and Combined Exercise on Metabolic Syndrome Parameters and Cardiovascular Risk Factors: A Systematic Review and Network Meta-Analysis, Reviews in Cardiovascular Medicine. (2021) 22, no. 4, 1523–1533, 10.31083/j.rcm2204156, 34957791. 34957791

[35] Owen N. , Sugiyama T. , Eakin E. E. , Gardiner P. A. , Tremblay M. S. , and Sallis J. F. , Adults’ Sedentary Behavior Determinants and Interventions, American Journal of Preventive Medicine. (2011) 41, no. 2, 189–196, 10.1016/j.amepre.2011.05.013, 2-s2.0-79960512967, 21767727. 21767727

[36] Tremblay M. S. , Aubert S. , Barnes J. D. , Saunders T. J. , Carson V. , Latimer-Cheung A. E. , Chastin S. F. M. , Altenburg T. M. , Chinapaw M. J. M. , and SBRN Terminology Consensus Project Participants , Sedentary Behavior Research Network (SBRN)—Terminology Consensus Project Process and Outcome, International Journal of Behavioral Nutrition and Physical Activity. (2017) 14, no. 1, 10.1186/s12966-017-0525-8, 2-s2.0-85020417774, 28599680. PMC5466781 28599680

[37] Wen C. P. and Wu X. , Stressing Harms of Physical Inactivity to Promote Exercise, Lancet. (2012) 380, no. 9838, 192–193, 10.1016/S0140-6736(12)60954-4, 2-s2.0-84864049285, 22818933. 22818933

[38] Katzmarzyk P. T. , Powell K. E. , Jakicic J. M. , Troiano R. P. , Piercy K. , Tennant B. , and 2018 Physical Activity Guidelines Advisory Committee , Sedentary Behavior and Health: Update From the 2018 Physical Activity Guidelines Advisory Committee, Medicine and Science in Sports and Exercise. (2019) 51, no. 6, 1227–1241, 10.1249/MSS.0000000000001935, 2-s2.0-85065973652, 31095080. 31095080 PMC6527341

[39] Guthold R. , Willumsen J. , and Bull F. C. , What Is Driving Gender Inequalities in Physical Activity Among Adolescents?, Journal of Sport and Health Science. (2022) 11, no. 4, 424–426, 10.1016/j.jshs.2022.02.003, 35217213. 35217213 PMC9338329

[40] The Lancet Public Health , Time to Tackle the Physical Activity Gender Gap, Lancet Public Health. (2019) 4, no. 8, e360, 10.1016/S2468-2667(19)30135-5, 2-s2.0-85069609113. 31345750

[41] Gulati M. , Black H. R. , Shaw L. J. , Arnsdorf M. F. , Bairey Merz C. N. , Lauer M. S. , Marwick T. H. , Pandey D. K. , Wicklund R. H. , and Thisted R. A. , The Prognostic Value of a Nomogram for Exercise Capacity in Women, New England Journal of Medicine. (2005) 353, no. 5, 468–475, 10.1056/NEJMoa044154, 2-s2.0-23044460445. 16079370

[42] Ji H. , Gulati M. , Huang T. Y. , Kwan A. C. , Ouyang D. , Ebinger J. E. , Casaletto K. , Moreau K. L. , Skali H. , and Cheng S. , Sex Differences in Association of Physical Activity With All-Cause and Cardiovascular Mortality, Journal of the American College of Cardiology. (2024) 83, no. 8, 783–793, 10.1016/j.jacc.2023.12.019, 38383092. 38383092 PMC10984219

[43] Sattelmair J. , Pertman J. , Ding E. L. , Kohl H. W. , Haskell W. , and Lee I. M. , Dose Response Between Physical Activity and Risk of Coronary Heart Disease: A Meta-Analysis, Circulation. (2011) 124, no. 7, 789–795, 10.1161/CIRCULATIONAHA.110.010710, 2-s2.0-80051944900. 21810663 PMC3158733

[44] Qian J. , Walkup M. P. , Chen S.-H. , Brubaker P. H. , Bond D. S. , Richey P. A. , Jakicic J. M. , Hu K. , Scheer F. A. J. L. , and Middelbeek R. J. W. , Association of Objectively Measured Timing of Physical Activity Bouts With Cardiovascular Health in Type 2 Diabetes, Diabetes Care. (2021) 44, no. 4, 1046–1054, 10.2337/dc20-2178. 33597215 PMC7985432

[45] Dastmalchi L. N. and Gulati M. , Age at Menarche and Cardiovascular Risk: A Moving Target of Risk Assessment in Women, European Journal of Preventive Cardiology. (2025) 32, no. 10, 867–869, 10.1093/eurjpc/zwaf318, 40468995. 40468995

[46] Piercy K. L. , Troiano R. P. , Ballard R. M. , Carlson S. A. , Fulton J. E. , Galuska D. A. , George S. M. , and Olson R. D. , The Physical Activity Guidelines for Americans, JAMA. (2018) 320, no. 19, 2020–2028, 10.1001/jama.2018.14854, 2-s2.0-85056407611. 30418471 PMC9582631

[47] Lavie C. J. , Ozemek C. , Carbone S. , Katzmarzyk P. T. , and Blair S. N. , Sedentary Behavior, Exercise, and Cardiovascular Health, Circulation Research. (2019) 124, no. 5, 799–815, 10.1161/CIRCRESAHA.118.312669, 2-s2.0-85064633214, 30817262. 30817262

[48] Polo-López A. , Calatayud J. , López-Bueno L. , Núñez-Cortés R. , Andersen L. L. , and López-Bueno R. , Dose-Response Association of an Accelerometer-Measured Physical Activity With All-Cause Mortality and Cardiovascular Disease Incidence: Prospective Cohort With 76,074 Participants, Progress in Cardiovascular Diseases. (2024) 87, 2–7, 10.1016/j.pcad.2024.10.004, 39389333. 39389333

[49] Garber C. E. , Blissmer B. , Deschenes M. R. , Franklin B. A. , Lamonte M. J. , Lee I. M. , Nieman D. C. , Swain D. P. , and American College of Sports Medicine , American College of Sports Medicine Position Stand. Quantity and Quality of Exercise for Developing and Maintaining Cardiorespiratory, Musculoskeletal, and Neuromotor Fitness in Apparently Healthy Adults: Guidance for Prescribing Exercise, Medicine and Science in Sports and Exercise. (2011) 43, no. 7, 1334–1359, 10.1249/MSS.0b013e318213fefb, 2-s2.0-79959902659, 21694556. 21694556

[50] Chen T. J. , Whitfield G. P. , Watson K. B. , Fulton J. E. , Ussery E. N. , Hyde E. T. , and Rose K. , Awareness and Knowledge of the Physical Activity Guidelines for Americans, 2nd Edition, Journal of Physical Activity & Health. (2023) 20, no. 8, 742–751, 10.1123/jpah.2022-0478, 37172953. 37172953 PMC10527333

[51] Wong N. D. and Sattar N. , Cardiovascular Risk in Diabetes Mellitus: Epidemiology, Assessment and Prevention, Nature Reviews. Cardiology. (2023) 20, no. 10, 685–695, 10.1038/s41569-023-00877-z, 37193856. 37193856

[52] Ma C. X. , Ma X. N. , Guan C. H. , Li Y. D. , Mauricio D. , and Fu S. B. , Cardiovascular Disease in Type 2 Diabetes Mellitus: Progress Toward Personalized Management, Cardiovascular Diabetology. (2022) 21, no. 1, 10.1186/s12933-022-01516-6, 35568946. PMC9107726 35568946

[53] Kodama S. , Tanaka S. , Heianza Y. , Fujihara K. , Horikawa C. , Shimano H. , Saito K. , Yamada N. , Ohashi Y. , and Sone H. , Association Between Physical Activity and Risk of All-Cause Mortality and Cardiovascular Disease in Patients With Diabetes: A Meta-Analysis, Diabetes Care. (2013) 36, no. 2, 471–479, 10.2337/dc12-0783, 2-s2.0-84873816641. 23349151 PMC3554302

[54] Laaksonen D. E. , Lindström J. , Lakka T. A. , Eriksson J. G. , Niskanen L. , Wikström K. , Aunola S. , Keinänen-Kiukaanniemi S. , Laakso M. , Valle T. T. , Ilanne-Parikka P. , Louheranta A. , Hämäläinen H. , Rastas M. , Salminen V. , Cepaitis Z. , Hakumäki M. , Kaikkonen H. , Härkönen P. , Sundvall J. , Tuomilehto J. , Uusitupa M. , and Finnish diabetes prevention study , Physical Activity in the Prevention of Type 2 Diabetes: The Finnish Diabetes Prevention Study, Diabetes. (2005) 54, no. 1, 158–165, 15616024. 15616024 10.2337/diabetes.54.1.158

[55] Chow L. S. , Odegaard A. O. , Bosch T. A. , Bantle A. E. , Wang Q. , Hughes J. , Carnethon M. , Ingram K. H. , Durant N. , Lewis C. E. , Ryder J. , Shay C. M. , Kelly A. S. , and Schreiner P. J. , Twenty Year Fitness Trends in Young Adults and Incidence of Prediabetes and Diabetes: The CARDIA Study, Diabetologia. (2016) 59, no. 8, 1659–1665, 10.1007/s00125-016-3969-5, 2-s2.0-84968585963, 27181604. 27181604 PMC4930716

[56] Crandall J. P. , Knowler W. C. , Kahn S. E. , Marrero D. , Florez J. C. , Bray G. A. , Haffner S. M. , Hoskin M. , and Nathan D. M. , The Prevention of Type 2 Diabetes, Nature Clinical Practice. Endocrinology & Metabolism. (2008) 4, no. 7, 382–393, 10.1038/ncpendmet0843, 2-s2.0-45849132668. PMC2573045 18493227

[57] Kriska A. M. , Rockette-Wagner B. , Edelstein S. L. , Bray G. A. , Delahanty L. M. , Hoskin M. A. , Horton E. S. , Venditti E. M. , Knowler W. C. , and DPP Research Group , The Impact of Physical Activity on the Prevention of Type 2 Diabetes: Evidence and Lessons Learned From the Diabetes Prevention Program, a Long-Standing Clinical Trial Incorporating Subjective and Objective Activity Measures, Diabetes Care. (2021) 44, no. 1, 43–49, 10.2337/dc20-1129, 33444158. 33444158 PMC7783946

[58] Tarp J. , Støle A. P. , Blond K. , and Grøntved A. , Cardiorespiratory Fitness, Muscular Strength and Risk of Type 2 Diabetes: A Systematic Review and Meta-Analysis, Diabetologia. (2019) 62, no. 7, 1129–1142, 10.1007/s00125-019-4867-4, 2-s2.0-85064599359, 31011778. 31011778 PMC6560020

[59] Kivimäki M. , Singh-Manoux A. , Pentti J. , Sabia S. , Nyberg S. T. , Alfredsson L. , Goldberg M. , Knutsson A. , Koskenvuo M. , Koskinen A. , Kouvonen A. , Nordin M. , Oksanen T. , Strandberg T. , Suominen S. B. , Theorell T. , Vahtera J. , Vaananen A. , Virtanen M. , Westerholm P. , Westerlund H. , Zins M. , Seshadri S. , Batty G. D. , Sipilä P. N. , Shipley M. J. , Lindbohm J. V. , Ferrie J. E. , and Jokela M. , Physical Inactivity, Cardiometabolic Disease, and Risk of Dementia: An Individual-Participant Meta-Analysis, BMJ. (2019) 365, l1495, 10.1136/bmj.l1495, 2-s2.0-85064695071. 30995986 PMC6468884

[60] Sadarangani K. P. , Hamer M. , Mindell J. S. , Coombs N. A. , and Stamatakis E. , Physical Activity and Risk of All-Cause and Cardiovascular Disease Mortality in Diabetic Adults From Great Britain: Pooled Analysis of 10 Population-Based Cohorts, Diabetes Care. (2014) 37, no. 4, 1016–1023, 10.2337/dc13-1816, 2-s2.0-84897857402, 24652727. 24652727

[61] Gregg E. W. , Jakicic J. M. , Blackburn G. , Bloomquist P. , Bray G. A. , Clark J. M. , Coday M. , Curtis J. M. , Egan C. , Evans M. , Foreyt J. P. , Foster G. D. , Hazuda H. P. , Hill J. O. , Horton E. S. , Hubbard V. S. , Jeffery R. W. , Johnson K. C. , Kitabchi A. E. , Knowler W. C. , Kriska A. M. , Lang W. , Lewis C. E. , Montez M. G. , Nathan D. M. , Neiberg R. H. , Patricio J. , Peters A. L. , Pi-Sunyer X. , Pownall H. , Redmon B. , Regensteiner J. G. , Rejeski J. , Ribisl P. M. , Safford M. M. , Stewart K. , Trence D. , Wadden T. A. , Wing R. R. , and Yanovski S. Z. , Association of the Magnitude of Weight Loss and Changes in Physical Fitness With Long-Term Cardiovascular Disease Outcomes in Overweight or Obese people With Type 2 Diabetes: A Post-Hoc Analysis of the Look AHEAD Randomised Clinical Trial, Lancet Diabetes and Endocrinology. (2016) 4, no. 11, 913–921, 10.1016/S2213-8587(16)30162-0, 2-s2.0-84993315699, 27595918. 27595918 PMC5094846

[62] Anand V. , Garg S. , Garg J. , Bano S. , and Pritzker M. , Impact of Exercise Training on Cardiac Function Among Patients With Type 2 Diabetes: A Systematic Review and Meta-Analysis, Journal of Cardiopulmonary Rehabilitation and Prevention. (2018) 38, no. 6, 358–365, 10.1097/HCR.0000000000000353, 2-s2.0-85055618828, 30142130. 30142130

[63] Teck J. , Diabetes-Associated Comorbidities, Primary Care. (2022) 49, no. 2, 275–286, 10.1016/j.pop.2021.11.004, 35595482. 35595482

[64] Oppert J. M. , Bellicha A. , van Baak M. A. , Battista F. , Beaulieu K. , Blundell J. E. , Carraça E. V. , Encantado J. , Ermolao A. , Pramono A. , Farpour Lambert N. , Woodward E. , Dicker D. , and Busetto L. , Exercise Training in the Management of Overweight and Obesity in Adults: Synthesis of the Evidence and Recommendations From the European Association for the Study of Obesity Physical Activity Working Group, Obesity Reviews. (2021) 22, no. Supplement 4, e13273, 10.1111/obr.13273. 34076949 PMC8365734

[65] Authors/Task Force Members , ESC Committee for Practice Guidelines (CPG) , and ESC National Cardiac Societies , 2019 ESC/EAS Guidelines for the Management of Dyslipidaemias: Lipid Modification to Reduce Cardiovascular Risk, Atherosclerosis. 2019, no. 290, 140–205. 10.1016/j.atherosclerosis.2019.08.014 31591002

[66] Kidney Disease: Improving Global Outcomes (KDIGO) CKD Work Group , KDIGO 2024 Clinical Practice Guideline for the Evaluation and Management of Chronic Kidney Disease, Kidney International. (2024) 105, no. 4S, S117–S314, 10.1016/j.kint.2023.10.018. 38490803

[67] Abdelhay O. , Altamimi M. , Abdelhay Q. , Manajrah M. , Tourkmani A. M. , Altamimi M. , and Altamimi T. , Perceived Barriers to Physical Activity and Their Predictors Among Adults in the Central Region in Saudi Arabia: Gender Differences and Cultural Aspects, PLoS One. (2025) 20, no. 2, e0318798, 10.1371/journal.pone.0318798, 39919050. 39919050 PMC11805373

[68] Alghafri T. , Alharthi S. M. , Al Farsi Y. M. , Bannerman E. , Craigie A. M. , and Anderson A. S. , Perceived Barriers to Leisure Time Physical Activity in Adults With Type 2 Diabetes Attending Primary Healthcare in Oman: A Cross-Sectional Survey, BMJ Open. (2017) 7, no. 11, e016946, 10.1136/bmjopen-2017-016946, 2-s2.0-85040693489, 29102987. PMC5722082 29102987

[69] Salmi L. V. , Hasanen E. , Simula M. , Virmasalo I. , and Muukkonen P. , Perceived Barriers to Physical Activity in the Social Spaces of Low Socioeconomic Status Suburbs, Wellbeing, Space and Society. (2023) 5, 100164, 10.1016/j.wss.2023.100164.

[70] Overcoming Barriers to Physical Activity, Physical Activity Basics 2025, http://www.cdc.gov/physical-activity-basics/overcoming-barriers/index.html.

[71] Breaking Down Barriers to Fitness, January 2024, https://www.heart.org/en/healthy-living/fitness/getting-active/breaking-down-barriers-to-fitness.

[72] Exercise and Heart Health. Healthy Living, 2019, https://www.cardiosmart.org/topics/healthy-living/move-more.

[73] Lindsay Smith G. , Banting L. , Eime R. , O’Sullivan G. , and van Uffelen J. G. Z. , The Association Between Social Support and Physical Activity in Older Adults: A Systematic Review, International Journal of Behavioral Nutrition and Physical Activity. (2017) 14, no. 1, 10.1186/s12966-017-0509-8, 2-s2.0-85018286152, 28449673. PMC5408452 28449673

[74] Mahdaviani B. , Soleimani Z. , Selk Ghaffari M. , Pourgharib Shahi M. H. , Masoumi S. , and Kordi R. , Barriers to Physical Activity in the Iranian Population: Findings From the STEPwise Surveillance 2021, BMC Public Health. (2024) 24, no. 1, 10.1186/s12889-024-20134-3, 39334036. PMC11438118 39334036

[75] Richards E. A. and Woodcox S. , Barriers and Motivators to Physical Activity Prior to Starting a Community-Based Walking Program, International Journal of Environmental Research and Public Health. (2021) 18, no. 20, 10659, 10.3390/ijerph182010659. 34682405 PMC8535237

[76] Bytyci Katanolli A. , Probst Hensch N. , Obas K. A. , Gerold J. , Zahorka M. , Jerliu N. , Ramadani Q. , Fota N. , and Merten S. , Perceived Barriers to Physical Activity Behaviour Among Patients With Diabetes and Hypertension in Kosovo: A Qualitative Study, BMC Primary Care. (2022) 23, no. 1, 10.1186/s12875-022-01866-w, 36180857. PMC9523175 36180857

[77] Adamson B. C. , Kinnett Hopkins D. , Athari Anaraki N. , and Sebastião E. , The Experiences of Inaccessibility and Ableism Related to Physical Activity: A Photo Elicitation Study Among Individuals With Multiple Sclerosis, Disability and Rehabilitation. (2022) 44, no. 12, 2648–2659, 10.1080/09638288.2020.1844315. 33174442

[78] Metsios G. S. , Fenton S. A. M. , Tzika K. , Moe R. H. , Fragoulis G. E. , Vliet Vlieland T. P. M. , Nikiphorou E. , Van den Ende C. H. M. , Fatouros I. , van der Esch M. , Niedermann K. , Stavropoulos Kalinoglou A. , Veldhuijzen van Zanten J. J. C. S. , Brodin N. , O’Brien C. M. , Koutedakis Y. , Kennedy N. , Swinnen T. W. , Bostrom C. , and Kitas G. D. , Barriers and Facilitators for Physical Activity in Rheumatic and Musculoskeletal Disease: A European-Based Survey, Clinical Rheumatology. (2023) 42, no. 7, 1897–1902, 10.1007/s10067-023-06518-7, 36877304. 36877304

[79] Anens E. , Emtner M. , and Hellström K. , Exploratory Study of Physical Activity in Persons With Charcot-Marie-Tooth Disease, Archives of Physical Medicine and Rehabilitation. (2015) 96, no. 2, 260–268, 10.1016/j.apmr.2014.09.013, 2-s2.0-84921434982, 25286435. 25286435

[80] Marashi M. Y. , Nicholson E. , Ogrodnik M. , Fenesi B. , and Heisz J. J. , A Mental Health Paradox: Mental Health Was Both a Motivator and Barrier to Physical Activity During the COVID-19 Pandemic, PLoS One. (2021) 16, no. 4, e0239244, 10.1371/journal.pone.0239244, 33793550. 33793550 PMC8016471

